# Impact of mental disorders and chronic physical conditions on quality-adjusted life years in Singapore

**DOI:** 10.1038/s41598-020-59604-0

**Published:** 2020-02-14

**Authors:** Edimansyah Abdin, Siow Ann Chong, Janhavi Ajit Vaingankar, Saleha Shafie, Swapna Verma, Nan Luo, Kelvin Bryan Tan, Lyn James, Derrick Heng, Mythily Subramaniam

**Affiliations:** 1 Research Division, Institute of Mental Health, Singapore, Singapore; 2 Early Psychosis Intervention Programme, Institute of Mental health, Singapore, Singapore; 3 Saw Swee Hock School of Public Health, National University of Singapore, Singapore, Singapore; 4 Ministry of Health, Singapore, Singapore

**Keywords:** Anxiety, Depression, Obsessive compulsive disorder, Epidemiology, Quality of life

## Abstract

The current study aims to evaluate the burden of disease in Singapore by estimating the quality-adjusted life years (QALYs) lost due to mental disorders and chronic physical conditions. The second Singapore Mental Health Study (SMHS-2016) was conducted in 2016 among 6126 respondents aged 18 years and above. The World Health Organization Composite International Diagnostic Interview version 3.0 (WHO-CIDI 3.0) and a modified version of the CIDI chronic medical disorders checklist were used to assess the 12-month diagnoses of mental and chronic physical disorders while the SF-6D scores derived from the 12-item Short Form Health Survey instrument was used to estimate the QALYs lost. The mean SF-6D score in this population was 0.87. The largest reduction in SF-6D scores among people with mental disorders was observed in Generalized Anxiety Disorder (GAD), followed by Major Depressive Disorder (MDD), alcohol abuse, bipolar disorder and Obsessive Compulsive Disorder (OCD) while the largest reduction in SF-6D score among people with chronic physical conditions was observed in ulcer, followed by lung disease, chronic pain and cardiovascular disease. At the population level, chronic pain was associated with the greatest QALY loss followed by MDD (14,204 and 6,889 respectively). Lung disease was associated with the smallest QALY loss (376). These findings highlight chronic pain, MDD, OCD, cardiovascular disease and GAD as the five leading contributors of QALYs lost in the general population which deserve prioritisation in public health prevention programmes.

## Introduction

Between 1990 and 2017, the decline in mortality rates has been associated globally with increasing life span, and an ageing population which has translated into an increase in the magnitude of the non-fatal disease burden^1^. The Global Burden of Disease Study (GBD)^1,2^ has reported that the leading contributors of years lived with disability (YLDs) are related to pain - low back pain, headache disorders, mental - depressive disorders, and metabolic disorders-diabetes^1^. In parallel with the transformation of the healthcare model towards a holistic person-centred approach^3–5^ and transition of focus from infectious diseases to non-communicable diseases^6,7^, quality-adjusted life years (QALYs)^8^ has increasingly become a valuable tool to estimate the burden of disease in the general population^9–11^. A QALY is a summary measure that combines the length of survival of an individual and the health-related quality of life^8^ by placing a value on time spent in different health states^12^. The value is reflective of the preference weight that society gives for different health states based on their own health preference^12^. For example, a person with full health has a utility score value of 1 while a health state equivalent to being dead is given a value of 0^12,13^.

Several studies have been conducted to estimate QALY losses attributed to chronic medical conditions which includes both physical and psychiatric disorders^9,14,15^. The QALY losses in the population can be calculated as a product of the marginal effect of each disorder, i.e., change in the health-related quality of life associated with a disorder multiplied with its prevalence in the general population^15,16^. The value is interpreted as the annual loss in QALYs resulting from the disorder, without considering mortality^9,14,15^. This method is commonly used to measure the burden of disease in the general population so that health services and initiatives can be planned to target the relevant chronic disorder that has a higher impact on the individual and the society.

Singapore, a country in South-East Asia has a total population of about six million. The Chinese (74.4%) form the majority of the population, followed by Malays (13.4%), Indians (9.0%) and those from other ethnic groups (3.2%)^17^. As the population in Singapore is rapidly ageing with a growing chronic disease burden, data on current disease burden in terms of QALY losses due to chronic physical and mental disorders are important tools for monitoring the burden of these conditions on the population^9^. An epidemiological study conducted in 2010 - the Singapore Mental Health Study (hereafter referred to as SMHS-2010) had shown that chronic pain conditions, hypertension, and major depressive disorders (MDD) were the largest contributor to QALY losses in Singapore^9^. It was also found that the impact of the two mood disorders - MDD and bipolar disorder, as well as one of the anxiety disorders - obsessive compulsive disorder (OCD), examined in the study were associated with significantly larger QALY losses than the impact of any chronic physical condition at an individual level^9^. Recently, the second SMHS was carried out in 2016 (hereafter referred to as SMHS-2016)^18^. Although previous studies have investigated the impact of mental and physical disorders on QALY in the general population^9^, little is known about the change in the prevalence of these conditions over the years and its impact on QALYs. Hence, the current study aims to estimate QALYs lost due to mental disorders and chronic physical conditions in Singapore using the recent data from the second SMHS-2016.

## Methods

### Sample

Data were obtained from the SMHS-2016 survey^18^ - a nationally representative cross-sectional survey conducted among resident adults aged 18 years and above in Singapore. The study design and characteristics of the sample of this survey have been described in detail elsewhere^18^. In brief, the study applied a disproportionate stratified random sampling design. Over a period of 1-year, face-to-face interviews were conducted with the participants. The respondents received an inconvenience fee of $60 for their participation in the survey.

### SF-6D

We used the SF-12^19^, a multidimensional health classification system assessing the six health domains of physical functioning, role limitation, social functioning, pain, mental health, and vitality, across 4–6 levels for each domain^19,20^. This instrument is based on 11 items from the 36-item Short Form Health Survey (SF-36)^21^ or 7 of the 12-item Short Form Health Survey (SF-12)^13,19^. This instrument has been widely used to generate utility values and is psychometrically sound in measuring health-related quality of life outcomes in both general and specific populations^13,21^. The SF-6D health state is defined by selecting 1 level from each domain, which results in a total of 18,000 possible health states^19,21^. The SF-6D scoring algorithm was developed using the standard gamble (SG) method from a sample of 249 SF-6D health states from a representative sample of the UK population^19,21^. Utility scores generated by the SF-6D range from 0.29 to 1.00, with 1.00 representing full health and 0.29 representing the worst possible health state defined by the SF-6D (i.e., all domains being at the worst level)^19,21^. The utility scores derived from English and Chinese versions of the SF-6D have been demonstrated to be equivalent in Singapore’s multi-ethnic general population^22^ and the instrument performs well in patients with mental illnesses^23^.

### Mental disorder

Mental disorders were assessed using the World Health Organization Composite International Diagnostic Interview (WHO)-CIDI), a fully structured diagnostic interview to assess mental disorders and their treatment^24^. Only selected diagnostic modules for 12-month prevalence of mood disorders (major depressive disorder (MDD), dysthymia and bipolar disorder), anxiety disorders (generalized anxiety disorder (GAD) and obsessive-compulsive disorder (OCD)), and alcohol abuse and dependence were included^18^. Diagnoses of mental disorders were based on the Diagnostic and Statistical Manual of Mental Disorders, Fourth Edition (DSM-IV) criteria^25^. CIDI hierarchy rules were applied to all diagnoses^18^.

### Chronic physical disorders

Information on chronic physical disorders was obtained using a modified version of the CIDI checklist of chronic medical disorders^26^. The question was read as ‘I’m going to read to you a list of health problems some people have. Has a doctor ever told you that you have any of the following…’ This was followed by a list of chronic physical disorders which were considered prevalent in Singapore’s population^26^. Eighteen individual disorders included in the current survey were re-classified into 11 types of common chronic physical conditions: (1) asthma, (2) diabetes, (3) hypertension and high blood pressure, (4) chronic pain (arthritis or rheumatism, back problems including disk or spine, migraine headaches), (5) cancer, (6) cardiovascular disease (stroke or major paralysis, heart attack, coronary heart disease, angina, congestive heart failure or other heart disease), (7) ulcer and chronic inflamed bowel disease (stomach ulcer, chronic inflamed bowel, enteritis, or colitis), (8) thyroid disease, (9) neurological condition (epilepsy, convulsions, fainting spells, or Parkinson’s disease), (10) chronic lung disease (chronic bronchitis or emphysema (excluding asthma)), and, (11) hyperlipidaemia. Those who gave a positive answer to the list of chronic physical disorders were routed to the following question “Did you receive any treatment for it at any time during the past 12-month.” Those who answered positively to both the questions were then identified as having a chronic physical condition for the past 12-months in this study^9,26^.

### Socio-demographic data

Data on gender, age group (18–34 years, 35–49 years, 50–64 years, and 65 years old and above), ethnicity (Chinese, Malay, Indian, and Others), marital status (never married, married, divorced/separated or widowed), educational level (primary and below, secondary, vocational /ITE, pre-university/junior college/diploma, and university), employment status (employed, unemployed and economically inactive i.e., students, homemakers and retirees) and average household income per month (below Singapore Dollar (SGD)2000, SGD2,000-SGD3,999, SGD4,000 - SGD5999, SGD6,000 - SGD9,999, SGD10,000 and above) in the past 12 months were collected.

### Statistical analysis

All estimates were weighted to adjust for over sampling, non-response and post-stratified for age and ethnicity distributions between the survey sample and the Singapore resident population^18^. Descriptive analyses were performed to describe socio-demographic profiles and mean SF-6D score according to different levels of sociodemographic factors in the study population. Multiple linear regression analyses were used to estimate the impact of 12-month chronic physical conditions and mental disorders on SF-6D score after controlling for all chronic physical conditions, mental disorders, and sociodemographic variables including age group, gender, ethnicity, marital status, education, employment and income status. The reduction in SF-6D scores due to each 12-month mental disorder and 12-month chronic physical condition was defined as the difference in SF-6D scores between persons with and without such conditions based on marginal effects of multivariate linear regression model. The QALY loss in the population associated with each health condition was estimated by multiplying the marginal effects from regression model by the prevalence of the disorder. This is interpreted as the annual loss in QALYs resulting from the disorder, without considering mortality^14,15^. A separate analysis by subpopulation at different age groups were also performed. Standard errors (SE) and significance tests were estimated using the Taylor series linearization method. All statistically significant differences were evaluated at the 0.05 level using two-sided tests.

### Ethical standards

The authors assert that all procedures contributing to this work comply with the ethical standards of the relevant national and institutional committees on human experimentation and with the Helsinki Declaration 1975, as revised in 2008. The study was approved by the National Healthcare Groups’ Domain Specific Review Board. Written informed consent was obtained from all participants and parents or legally acceptable representatives of those aged below 21 years.

## Results

### Socio-demographic characteristics of the sample

A total of 6126 adult Singapore residents aged 18 years and above were recruited, which yielded a response rate of 69.5%. A total of 6,113 respondents who completed the SF-12 were included in the current study. The sample comprised 50.5% female and 49.5% male respondents. Majority of the respondents were aged between 18 and 49 years (60%), of Chinese ethnicity (75.7%), employed (72%) and currently married (60%) (Table 1).

**Table 1:** Descriptive statistics of the sample and socio-demographic correlates of the SF-6D.

	Sample		SF-6D				
	N	%	Mean	SD	Multiple linear regression		
					Beta coefficient	95% CI	P value
**Age group**							
18–34	1706	30.5	0.862	0.095	Ref.		
35–49	1494	29.6	0.879	0.088	0.013	(0.0005,0.025)	0.042
50–64	1623	26.9	0.877	0.101	0.015	(0.002,0.029)	0.029
65+	1290	13.1	0.856	0.147	0.007	(−0.011,0.025)	0.420
**Gender**							
Female	3050	50.5	0.865	0.104			
Male	3063	49.5	0.875	0.100	0.01	(0.002,0.019)	0.011
**Ethnicity**							
Chinese	1780	75.7	0.869	0.062	Ref.		
Malay	1982	12.4	0.870	0.174	0.002	(−0.006,0.01)	0.621
Indian	1842	8.7	0.870	0.202	0.001	(−0.007,0.008)	0.817
Others	509	3.1	0.886	0.167	0.016	(0.005,0.027)	0.005
**Education**							
Primary and below	1183	16.3	0.864	0.120	0.001	(−0.014,0.016)	0.886
Secondary	1641	23	0.867	0.120	−0.005	(−0.018,0.009)	0.492
Pre-U/Junior college	304	6.1	0.862	0.093	−0.01	(−0.029,0.009)	0.313
Vocational institute/ITE	508	6.3	0.875	0.118	0.0004	(−0.016,0.017)	0.968
Diploma	1023	19	0.872	0.092	−0.003	(−0.014,0.009)	0.650
University	1454	29.4	0.875	0.082	Ref.		
**Employment**							
Employed	4052	72	0.878	0.090	Ref.		
Economically inactive	1710	22.8	0.859	0.122	−0.007	(−0.018,0.004)	0.211
Unemployed	350	5.2	0.813	0.149	−0.055	(−0.081,−0.03)	<0.001
**Marital status**							
Never married	1542	31	0.860	0.090	−0.007	(−0.019,0.004)	0.210
Married	3836	59.8	0.878	0.101	Ref.		
Divorced/separated	342	5.2	0.858	0.121	−0.016	(−0.036,0.003)	0.107
Widowed	393	4.1	0.844	0.153	−0.014	(−0.036,0.008)	0.201
**Household income per month (SGD)**							
<2000	1140	16.4	0.848	0.137	Ref.		
2000–3999	1328	20	0.871	0.110	0.018	(0.004,0.031)	0.009
4000–5999	1112	21.4	0.878	0.092	0.024	(0.01,0.037)	0.001
6000–9999	1003	21.8	0.868	0.093	0.012	(−0.003,0.027)	0.111
10000 and above	860	20.4	0.879	0.080	0.021	(0.005,0.036)	0.009

ITE = Institute Technology of Education; Pre-U = Pre University; SGD = Singapore Dollar.

### SF-6D scores by different levels of sociodemographic factors

The mean SF-6D score in this population was 0.87. The mean SF-6D scores varied significantly by age group, gender, employment, marital status, and income. After controlling for all socio-demographic characteristics, multiple linear regression analysis revealed that those aged 35 to 64 years (vs. 18 to 34 years), male gender (vs. female), employed (vs. unemployed) and higher monthly household income (vs. less than SGD2000) were significantly associated with higher SF-6D scores (Table 1).

### SF-6D scores by type of conditions

**Figure 1:**
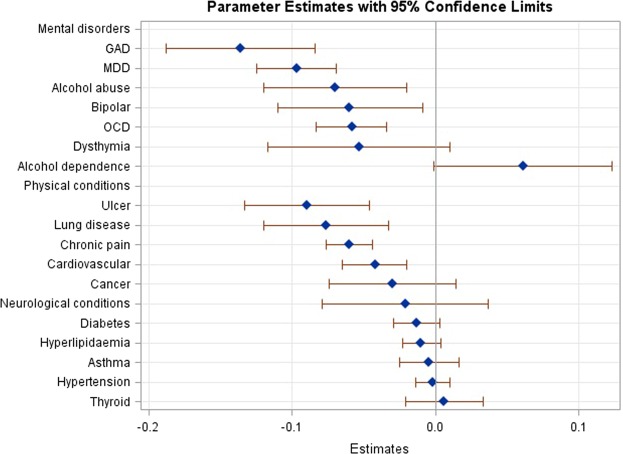
Marginal effects of each condition on SF-6D scores.

Table 2 presents the impact of mental disorders and chronic physical conditions on SF-6D scores. Of the eighteen conditions examined, five mental disorders (MDD, bipolar, OCD, GAD, and alcohol abuse) and four chronic physical conditions (chronic pain, cardiovascular, ulcers, and lung disease) were significantly associated with lower SF-6D scores. The largest reduction in SF-6D scores among people with mental disorders was observed in GAD, followed by MDD, alcohol abuse, bipolar disorder and OCD while the largest reduction in SF-6D scores among people with chronic physical conditions was observed among those with ulcer, followed by lung disease, chronic pain and cardiovascular disease (Fig. 1).

**Table 2:** Impact of type of mental disorders and chronic physical conditions on SF-6D scores.

	Beta coefficient	95% CI	P value
Mental disorders*			
GAD	−0.136	(−0.188,−0.084)	<0.001
MDD	−0.097	(−0.125,−0.069)	<0.001
Alcohol abuse	−0.07	(−0.12,−0.02)	0.006
Bipolar	−0.06	(−0.11,−0.009)	0.02
OCD	−0.058	(−0.083,−0.034)	<0.001
Dysthymia	−0.053	(−0.117,0.01)	0.101
Alcohol dependence	0.061	(−0.001,0.123)	0.051
Chronic physical conditions*			
Ulcer	−0.09	(−0.133,−0.046)	<0.001
Lung disease	−0.076	(−0.12,−0.033)	0.001
Chronic pain	−0.06	(−0.076,−0.044)	<0.001
Cardiovascular	−0.042	(−0.065,−0.02)	<0.001
Cancer	−0.03	(−0.074,0.014)	0.186
Neurological conditions	−0.021	(−0.079,0.037)	0.485
Diabetes	−0.013	(−0.029,0.003)	0.101
Hyperlipidaemia	−0.01	(−0.023,0.004)	0.166
Asthma	−0.005	(−0.025,0.016)	0.670
Hypertension	−0.002	(−0.014,0.01)	0.714
Thyroid	0.006	(−0.021,0.033)	0.640
**Age group**			
18–34	Ref.		
35–49	0.010	(−0.001,0.022)	0.076
50–64	0.015	(0.002,0.028)	0.024
65+	0.014	(−0.004,0.032)	0.136
Gender			
Female vs. Male	−0.009	(−0.016,−0.001)	0.023
**Ethnicity**			
Chinese	Ref.		
Malay	0.006	(−0.002,0.013)	0.154
Indian	0.007	(−0.001,0.014)	0.069
Others	0.017	(0.006,0.027)	0.002
**Marital status**			
Married	Ref.		
Never married	−0.006	(−0.016,0.005)	0.291
Divorced/separated	−0.008	(−0.026,0.009)	0.337
Widowed	−0.014	(−0.035,0.007)	0.197
**Education**			
University	Ref.		
Primary and below	0.006	(−0.008,0.021)	0.398
Secondary	−0.003	(−0.016,0.01)	0.604
Pre-U/Junior college	−0.004	(−0.022,0.013)	0.623
Vocational institute/ITE	0.002	(−0.013,0.018)	0.777
Diploma	−0.001	(−0.011,0.01)	0.915
**Employment**			
Employed	Ref.		
Economically inactive	−0.008	(−0.019,0.002)	0.131
Unemployed	−0.044	(−0.066,−0.021)	<0.001
**Household income per month (SGD)**			
<2000	Ref.		
2000–3999	0.014	(0.001,0.026)	0.033
4000–5999	0.020	(0.008,0.033)	0.002
6000–9999	0.011	(−0.003,0.025)	0.120
10000 and above	0.021	(0.006,0.035)	0.005

*The mental disorders and chronic physical conditions are rank ordered by the size of the beta coefficient.

### Population level QALY losses

Table 3 shows the annual QALY losses for the entire population that could be explained by each condition that reached statistical significance in the multivariate regression analyses. We found that chronic pain was associated with the greatest QALY loss at population level, followed by MDD (14,204 and 6,889 respectively). Lung disease was associated with the smallest QALY loss (376). We found chronic pain was also leading cause of QALY loss among those aged 35 years and above, while MDD had the greatest impact among those aged 18–34 years.

**Table 3:** Annual QALY losses for the entire population and at different age groups.

Condition	All			18–34 years			35–49 years			50–64 years			65+ years		
	%	Marginal effect	QALY*	%	Marginal effect	QALY	%	Marginal effect	QALY	%	Marginal effect	QALY	%	Marginal effect	QALY
Chronic pain	7.6	−0.060	−14204	5.3	−0.059	−2974	7.3	−0.040	−2655	8.1	−0.074	−4977	12.4	−0.067	−3361
MDD	2.3	−0.097	−6889	4.4	−0.083	−3481	1.8	−0.118	−1927	1.3	−0.132	−1402	0.5	−0.005	−11
OCD	2.9	−0.058	−5282	5.7	−0.042	−2267	3.1	−0.083	−2351	0.8	−0.074	−486	0.5	−0.044	−90
Cardiovascular	3.1	−0.042	−4122	0.3	−0.147	−481	1.1	−0.106	−1094	4.7	−0.020	−784	10.9	−0.012	−521
GAD	0.8	−0.136	−3561	1.1	−0.114	−1159	1.3	−0.160	−1853	0.5	−0.101	−436	0.0	−0.315	−57
Bipolar	0.9	−0.060	−1616	2.1	−0.073	−1439	0.4	0.040	164	0.4	−0.141	−451	0.0	−0.100	−12
Alcohol abuse	0.6	−0.070	−1224	1.1	−0.082	−863	0.5	−0.088	−429	0.2	0.020	39	0.0	0.000	0
Ulcer	0.4	−0.090	−1002	0.1	−0.066	−67	0.1	−0.166	−103	0.6	−0.115	−576	1.1	0.015	69
Lung	0.2	−0.076	−376	0.1	−0.093	−80	—	—	—	0.4	−0.072	−234	0.2	−0.046	−38

*Rank ordered by size of Annual loss of QALYs.

Note: Those estimates may over-estimate total loss of QALYs due to mental health because patients with multiple conditions do not experience the additive sum of the estimate utility losses.

## Discussion

This study has revealed that a number of mental and chronic physical conditions in Singapore were significantly associated with substantial QALY loss at the societal level and a significant decrease in health utility scores at an individual level. GAD had a greater impact on utility scores at the individual level as compared to other mental disorders and chronic physical conditions. It is possibly due to the fact that in our population, the proportion of those with GAD who reported ‘severe’ impact on functioning was highest among those with mental disorders, 7% of GAD cases were assessed to have had a severe disorder in the past one year using the Sheehan’s Disability Scale criteria^27^, while only 1% of bipolar cases were assessed to be severe. None of the alcohol abuse cases had severe disorder. Moreover, the marginal effect of GAD on utility scores was considered to have met clinically important different (CID) criterion. The CID can be defined as the smallest difference in the score that patients perceive as important that could lead a clinician to consider the change in the patient’s management^28^. In the current study, we adopted CID as additional information beyond the p - value in order to interpret the meaningfulness of the marginal effect of each disorder on utility scores from the patient’s perspective. Previous studies have reported that the CID values for SF-6D as 0.051 using an anchor-based method and the range as 0.01 to 0.48 using a distribution-based method^29^. It seems that different methods used can lead to different findings. Thus, if we use the anchor-based method to define CID, it seems that all mental disorders and only three chronic physical conditions can be considered as clinically important. However, if the lower range (0.01) of the distribution-based method is used, a number of physical conditions can be considered as achieving CID.

The finding that GAD had the highest impact on utility scores in the SMHS-2016 came as a surprise in light of the fact that GAD was associated with a smaller and insignificant reduction in health utility scores in the SMHS 2010^9^. The changes in the magnitude of the impact of GAD on utility scores between 2010 and 2016 are not easy to explain, because many factors may have played a significant role between these two time-points. It is possible that a significant increase in the lifetime (0.9% to 1.6%, p = 0.005) and 12-month (0.4% vs. 0.8%, p = 0.033) prevalence of GAD between SMHS 2010 and SMHS 2016^18^ may be associated with a substantial impact of GAD on the person’s physical and mental health^30,31^. It is also possible that the changes are due to the different instruments employed in the two surveys as the utility scores were measured based on EQ-5D in the SMHS-2010.

MDD was the second largest contributor to the reduction in utility scores at an individual-level after GAD. It was also the second largest contributor to the loss of QALY at the population level after chronic pain and the leading cause of QALY loss among younger age groups. This finding is partly consistent with our previous study which reported MDD as the largest contributor to the reduction of HRQoL at individual-level in SMHS 2010^9^. Higher prevalence of MDD in the general population^18,32^ with significantly impaired role functioning as well as increased days out of role could explain the significant impact of MDD on health utilities and QALYs^32^.

When we multiplied the marginal effects of each condition on utility scores with the prevalence, chronic pain was associated with the largest loss of QALYs in our population. Although the impact of GAD and MDD on utility scores was much higher than chronic pain at an individual level, chronic pain remained associated with the largest QALY loss at the societal level. This finding could be explained due to the higher prevalence of chronic pain in our population, which was almost 9 and 3 times higher than GAD and MDD, respectively. In this study, the prevalence of chronic pain was 7.6%, while the prevalence of GAD and MDD was 0.8% and 2.3%, respectively. To ensure comparability with previous studies, the current study defined chronic pain as those who experienced migraine, arthritis or rheumatism and back problems including “disc or spine problems”. The greater impact of chronic pain on QALYs at societal level was in line with recent epidemiological data that has shown that low back pain was the strongest contributor of non-fatal loss in terms of YLD globally^1^.

After controlling for sociodemographic factors, mental disorders and chronic physical conditions in multiple linear regression model, we found younger age, female gender, unemployment, and lower income were significantly associated with lower utility scores. Our findings are consistent with local data which found that the prevalence of mental disorders was higher among those of younger age^18^ and mental disorders represented the largest single contributor to the disease burden of disability-adjusted life years for Singaporeans between the ages of 10 and 34 years^33^. In Australia too mental disorders were found to be the leading cause of burden among those belonging to the younger age group followed by neonatal causes and unintentional injuries^34^. These evidence support the finding that the burden of mental disorders among those belonging to the younger age group is significant. Hence, initiatives to promote mental wellbeing and development of effective treatment strategies to improve young people’s mental health are needed.

In line with this, Sagayadevan *et al*.^35^ also found younger age and unemployment to be significantly associated with lower quality of life among local psychiatric outpatients. Similar findings were also reported among patients with mental disorders^36^ as well as in population-based studies conducted in other countries^37–39^. However, findings on the relationship between age and quality of life between SMHS 2010 and SMHS 2016 were mixed. In the SMHS 2010, those belonging to the younger age-group were significantly associated with a higher quality of life while these findings were reversed in 2016. Those in the younger age group in the current cohort may have been more vulnerable to economic and psychological stresses as compared to the previous cohort; however this needs further research.

The study has some limitations that need to be acknowledged. Firstly, the study used self-report to assess chronic physical conditions, and administrative data were not used for confirmation. A study by Knight *et al*. has shown that the chronic physical conditions checklist provides useful and accurate information about both treated and untreated chronic conditions^40^. Ye *et al*. have similarly reported that the self-reporting of chronic physical conditions provided information similar to that available from medical records^41^. Secondly, cross-sectional studies cannot ascertain causality, so longitudinal research is warranted. These limitations notwithstanding, this study was conducted on a nationally representative multi-ethnic population. The study had a good response rate, making the estimates highly generalizable to the multi-ethnic local population. All field interviewers were trained and stringent quality control was implemented throughout the study to ensure the data is reliable and valid. In all, 66 field interviewers were recruited for the study. All field interviewers underwent a structured training programme over a three-week period and were evaluated individually on all study- related procedures before being allowed to conduct the interviews in the field. The core research team members underwent training and were certified by the WMH-CIDI Training and Research Centre at the University of Michigan, USA. Stringent quality control measures were implemented throughout the study. For example, the number of completed interviews and time taken for each interview by each interviewer was closely tracked, on average two direct observation of each interviewer’s actual interviews at respondent’s household was conducted by core research team members, as well as 20% of completed interviews were randomly selected for verification via telephone calls/home visits to detect any falsification of the data.

In conclusion, the current study provides important evidence of the reduction of quality-adjusted life years in people with mental disorders and chronic physical conditions in Singapore. These findings highlight chronic pain, MDD, OCD, cardiovascular disease and GAD as five leading contributors of QALYs lost in the general population, which deserve prioritisation in public health prevention programmes.
